# Slower recovery of outpatient clinics than inpatient services for stroke and other neurological diseases after COVID‐19 Pandemic

**DOI:** 10.1111/cns.13459

**Published:** 2020-10-15

**Authors:** Jing Zhao, Yong Wang, Marc Fisher, Renyu Liu

**Affiliations:** ^1^ Department of Neurology Minhang Hospital Fudan University Shanghai China; ^2^ Department of Neurology Beth Israel Deaconess Medical Center Harvard Medical School Boston MA USA; ^3^ Department of Anesthesiology and Critical Care Perelman School of Medicine at the University of Pennsylvania Philadelphia PA USA

**Keywords:** clinic, COVID‐19, global impact, hospitalization, stroke, stroke care

## Abstract

**Background:**

In this brief report, we investigated the impact of COVID‐19 on outpatient stroke clinics and inpatient services and their recovery process.

**Methods:**

We sent a survey to physicians worldwide through the network of the World Stroke Organization to investigate the impact of COVID‐19 on stroke clinics. To farther along in recovering from the outbreak, we reviewed stroke and other neurology outpatient clinic visits (approximately 50% were stroke related) and the number of inpatient services from December 2019 to July 2020 in a large neurology department in Shanghai, China, where there was no official city lockdown.

**Results:**

We received 112 valid survey responses from 46 countries, representing all continents except for Antarctica. Only seven of the survey responders (7/112, 6.3%) reported that they have kept their outpatient clinics open as usual, but they did exercise increased precautions for COVID‐19 by following recent guidelines regarding use of personal protective equipment and isolation techniques. The remainder of the respondents have either reduced outpatient clinic services or suspended outpatient clinics completely. Telephone consultation or telemedicine with video capability was used for new patients or follow‐ups, with limited in‐person evaluations when necessary. Outpatient clinic visits and inpatient services from a large tertiary hospital in China decreased dramatically during the peak period of the outbreak, but then rebounded back quickly following the partial or full recovery from the outbreak. Compared with the recovery process of inpatient services, outpatient clinic visits decreased faster and recovered much slower. This is consistent with our global survey data which indicates that some outpatient clinics have rescheduled their outpatient visits for 3 to 6 months.

**Conclusions:**

The COVID‐19 pandemic caused a significant drop of in‐person outpatient visits and inpatient services. Clinic visits recovered slower than inpatient services in stroke and other neurological diseases after the pandemic.

## Graphical abstract

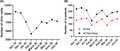

## 1 INTRODUCTION

We and others have reported recently that a significant drop of stroke admissions, evaluations, and related stroke care delivery occurred during the peak period of the novel coronavirus disease (COVID‐19) outbreak.^1^, ^2^, ^3^, ^4^, ^5^ As we continue monitoring the situation, we observed that many outpatient clinics have reduced or completely stopped in‐person outpatient visits, and there is possible significant difference in recovery process for clinic visits and inpatient services which may have a profound impact on disease management and prevention. In this brief report, we compared the impact of COVID‐19 pandemic on outpatient stroke clinics and inpatient services and their potential difference in recovery process.

## 2 MATERIALS AND METHODS

There is no patient or individual information involved in this study; therefore, no Internal Review Board approval was needed. We sent a survey, during the last two weeks of April 2020, through the network of the World Stroke Organization to ask two simple questions: “(a) Which country are you in? and (b) How are you handling outpatient stroke‐related visits now?”. Since China is the place where COVID‐19 was initially reported, and is therefore farther along in recovering from the outbreak, we reviewed stroke and other neurology outpatient clinic visits (of which approximately 50% were stroke related) and inpatient services from January 2019 to July 2020 from one large neurology department in Shanghai, China, where there had been no city lockdown.

### 2.1 Statistical analysis

This was a survey‐based study. All statistical analyses are descriptive, or reported as the actual numbers of observations. The graph is generated using Prism GraphPad (Version 8.2.1 for Windows 10, GraphPad Software, San Diego, CA). The recovery rate is calculated as follows: recovery rate = (number in the same month in 2020/number in the same month in 2019) × 100. A full recovery is defined as 100% as compared to the same period in 2019. Multiple comparisons for the differences among the recovery rates were performed using a chi‐square test. SPSS software (version 20.0, IBM Corporation, NY) was used for data analyses. Values of *P* < .05 are considered statistically significant.

## 3 RESULTS

We received 112 valid survey responses from 46 countries representing all continents except for Antarctica. The list of countries and the number of responses, and the summary of the responses are presented in Table 1. Only seven of the survey responders (7/112, 6.3%) reported that they have kept their outpatient clinics open as usual, but with increased precautions for COVID‐19 and following recent guidelines regarding personal protective equipment and isolation techniques. The remainder of the respondents reduced outpatient clinic services or suspended outpatient clinics completely. Telephone consultation or telemedicine with video capability was used for new patients or follow‐ups with limited in‐person evaluations when necessary. Two respondents reported that they have rescheduled their outpatient visits for 3 to 6 months. One reported from Australia that they are changing to telemedicine or phone follow‐up for approximately 80% of their patients. Transient ischemic attack patients are triaged and reviewed by a consultant, and telemedicine or phone visits are arranged with appropriate investigations. Inpatient stroke patients who are discharged are provided with detailed summaries for their family doctors to follow, to reduce postadmission reviews. New medical home visits are provided by some centers in the UK. One survey response from South Korea indicated that telemedicine was allowed during the pandemic, despite not being allowed prior to the pandemic.

**TABLE 1:** The impact on outpatient clinics from COVID‐19 pandemic and the usage of telemedicine

Where do you come from?		Number of answers	Impact on clinics	Telemedicine
Africa	Nigeria	2	Reduced/closed	No mention
Asia	China	1	Reduced	Telephone
	India	4	Reduced/closed	Telephone
	Japan	2	Open as usual with PPE	No mention
	Kyrgyzstan	1	Closed	No mention
	Malaysia	5	Reschedule 3‐6 mo	Virtual clinic/telephone
	Myanmar	1	Reduced/closed	No mention
	Nepal	1	Closed	No mention
	Oman	1	Closed	No mention
	Pakistan	1	Reduced	No mention
	Philippines	3	Reduced/closed	Email/SeriousMD App
	Saudi Arabia	1	Reduced	Video visit
	Singapore	1	Reduced	No mention
	South Korea	1	Mostly as usual	Newly allowed tele‐visit
	Sri Lanka	1	Reduced	Mainly teleconsults
	Thailand	1	Reduced or rescheduled	No mention
	Vietnam	1	Reduced/closed	Telemedicine
Europe	Belgium	4	Reduced/closed	Telephone
	Cornwall	1	Reduced/closed	Telephone/video call
	Croatia	1	Reduced/closed	Telephone
	France	2	Reduced	Telephone/telemedicine
	Germany	4	Normal/rescheduled	No mention
	Greece	4	Closed	Telephone/email
	Ireland	2	Reduced/closed	Phone/virtual clinic
	Italy	5	Normal/only for emergency	Telephone
	Lithuania	1	Closed	No mention
	North Macedonia	1	Open only for emergency	No mention
	Norway	1	Reduced	Telephone/video
	Portugal	2	Closed	No mention
	Russia	1	Closed	No mention
	Scotland	1	Closed	Telephone
	Slovakia	1	Closed	No mention
	Spain	5	Closed	Telephone
	Sweden	4	Reduced	Telephone/video
	United Kingdom	9	Limited visit/closed	Telephone/video
	Ukraine	3	Closed	Teleconsultations
North America	Canada	8	Closed	Telemedicine platform
	Costa Rica	1	Closed	Telephone
	United States	8	Limited visit/closed	Telehealth/video visits
South America	Argentina	1	Closed	Telemedicine
	Australia	3	Normal/reduced	Telemedicine
	Austria	2	Closed	Telephone
	Brazil	4	Reduced/closed	Telemedicine
	Chile	1	Closed	Telemedicine
	Colombia	3	Closed	Telemedicine
	Venezuela	1	Private emergency unit	No mention

Note

COVID‐19, novel coronavirus disease; App, Application; Teleconsults, telephone consult.

**FIGURE 1:**
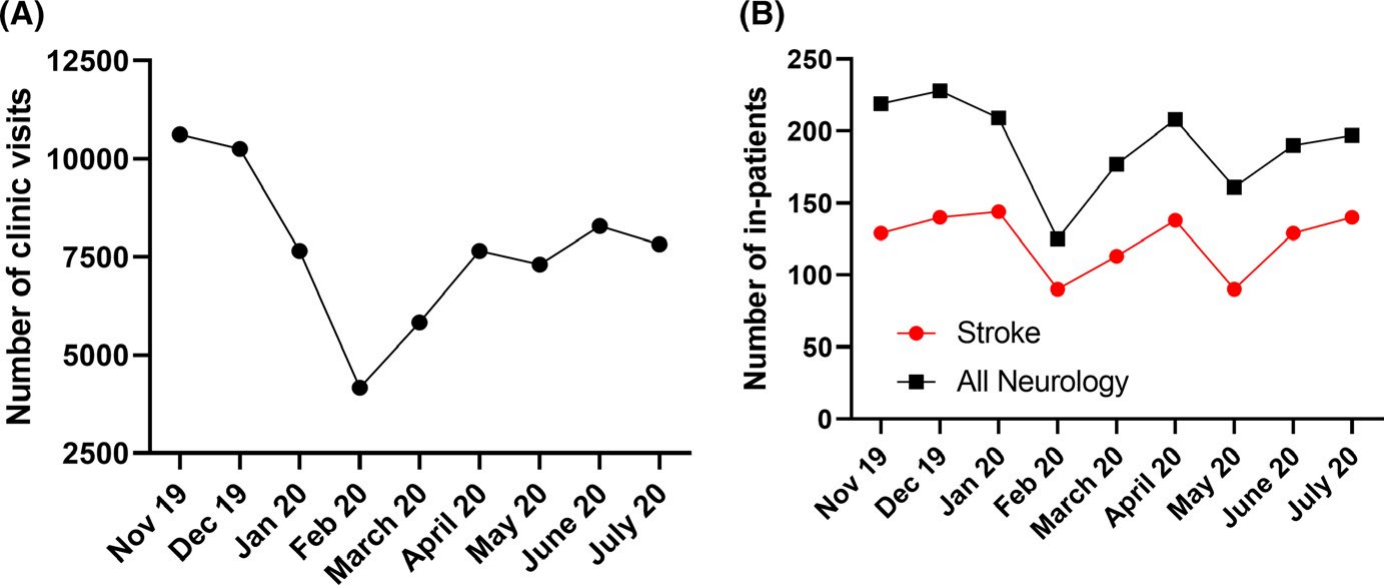
The changes in outpatient visits and inpatient services from the neurology department in a tertiary hospital in Shanghai, China, where there was no official city lockdown during the pandemic. A. It indicates the clinic visits from January 2019 to July 2020. Clinic visits dropped by 36.6% in February 2020 as compared to that in February in 2019, with a partial rebound as the effects of the pandemic waned; B. It indicates the inpatient services in stroke and all neurological diseases from January 2019 to July 2020. Inpatient services in stroke and all neurological diseases dropped by 27.4% and 23.8%, respectively, in February 2020 as compared to that in February in 2019 and then rose rapidly to a nearly normal status

As indicated in Figure 1A, outpatient clinic visits to a large tertiary hospital in China decreased dramatically by 36.6% during the peak period of the outbreak in February but then returned to 60.5%, 78.3%, 77.6%, 89.0%, and 78.8% in March, April, May, June, and July, respectively, as compared to the same month in 2019 (Table 2). This is consistent with our global survey data, which indicated that some of the outpatient clinics have rescheduled their outpatient visits for 3 to 6 months. As shown in Figure 1B, due to the fluctuation of outpatient clinic services, inpatient services in all neurological diseases dropped rapidly by 23.8% in February and in the following four months they returned to 65.8%, 82.2%, 64.7%, 78.5%, and 89.1%, respectively. Inpatient stroke admissions dropped rapidly by 27.4% in February and then rebounded quickly recovering to 103.9%, 92.2%, 66.1%, 90.0%, and 100% in March, April, May, June, and July, respectively, as compared to its pre‐COVID‐19 period. Other neurological disease leading to admission included vertebrobasilar insufficiency, epilepsy, Parkinson's disease, intracranial infection, headache, dementia, sleep disturbance, motor neuron disease, and spinal cord disease. Inpatient services experienced a decline in May 2020 similar to that in the peak period of COVID‐19 epidemic in the recovery process, which was related to the overly strict nucleic acid test mandate for inpatients and family members which was removed later on. The number and recovery rate of clinic visits and inpatient services in a large tertiary hospital in China were shown in Table 2. Compared with the recovery process of clinic visits, inpatient services recovered faster, and the number of visits did not fully recover at the end of this investigation.

**TABLE 2:** The number and recovery rate of clinic visits and inpatient services in a large tertiary hospital in Shanghai

Time (Mo, Y)	Clinic Visits		Inpatients (neurological diseases)		Stroke inpatients	
	Number of visits	Comparison with the same month last year	Number of patients	Comparison with the same month last year	Number of patients	Comparison with the same month last year
Jan 2019	9316.5	—	226	—	143	—
Feb 2019	6438	—	164	—	117	—
Mar 2019	9761.5	—	269	—	103	—
Apr 2019	9659.5	—	253	—	129	—
May 2019	9827	—	249	—	127	—
Jun 2019	9307	—	242	—	145	—
Jul 2019	9923	—	221	—	140	—
Aug 2019	9570.5	—	218	—	134	—
Sep 2019	7322	—	228	—	124	—
Oct 2019	7106	—	219	—	127	—
Nov 2019	9532	—	219	—	127	—
Dec 2019	9705	—	228	—	127	—
Jan 2020	7108.5	—	209	—	122	—
Feb 2020	4083.5	63.4%	125	76.2%*	85	72.6%
Mar 2020	5909.5	60.5%	177	65.8%	107	103.9%*
Apr 2020	7565	78.3%	208	82.2%	119	92.2%*
May 2020	7629.5	77.6%	161	64.7%*	84	66.1%*
Jun 2020	8286.5	89.0%	190	78.5%*	129	90.0%**^#^**
Jul 2020	7822.5	78.8%	197	89.1%*	140	100%*

* *P* < .05, compared with the changes in the clinic visits.

** *P* < .05, compared with the changes of inpatients for all neurological diseases.

## 4 DISCUSSION

Outpatient clinic visits play a critical role in patient education and stroke (and other neurological disease) prevention, and documenting patient characteristics related to stroke outcome.^6^ Secondary stroke prevention, including blood pressure, diabetes, and anticoagulation management, can reduce the incidence and severity of recurring stroke.^7^ The reduced service, or the total shutdown, of outpatient clinics for stroke patients can potentially lead to an increased incidence of stroke in the future. Fortunately, due to technology development, the availability of telemedicine was able to offset some of the negative impact from the pandemic related shutdown of outpatient clinics. Based on current clinical experience (personal experience), the adherence of patients to their scheduled telemedicine visits was actually improved compared to prior in‐person visits, with less patients missing their scheduled telemedicine appointments. Compared with the impact on inpatient services from the COVID‐19 epidemic, outpatient clinic visits decreased faster and recovered slower. Telemedicine could potentially play a significant role in the slower recovery of the outpatient clinic visit. It is possible that the negative psychological impact of COVID‐19 could potentially play a role in the slower recovery in the in‐person outpatient clinic visits. This is consistent with the observation that number of hospitalization dropped to a similar extent when a viral test is mandated despite there is no increase of COVID‐19 new cases.

The stroke‐related hospitalization recovered quite quickly since hospitalization for stroke therapy is necessary. This also reflects successful efforts from stroke neurologist in managing stroke patients in the era of COVID‐19 pandemic.

As hospitals and clinics are preparing to reopen to a full “routine” schedule, patients and accompanying person(s) will likely be required to wear masks to avoid hospital acquired COVID‐19, as we proposed recently as a “new normal.”^8^ Some hospitals and clinics have already implemented such a universal mask policy, for now and for as long as we still see COVID‐19 patients in the hospital or clinic settings.

The major limitation of this study is that we included only one center to indicate the trend of clinic visit and inpatient services recovery. We did not investigate the key factors that will drive the recovery and mechanisms of future clinic visits and inpatient services, so further studies are needed to investigate these issues. Another limitation is that the survey responses consist of those from only one center in some of the countries, which may not reflect the whole picture of the impact COVID‐19 has had on stroke clinics in these countries.

In conclusion, the COVID‐19 pandemic caused a significant drop of in‐person outpatient visits and inpatient services. The recovery process of clinic visits and inpatient services was different, and clinic visits recovered slower than inpatient services in stroke and other neurological diseases after the pandemic.

## CONFLICT OF INTEREST

None of the authors have any conflicts to report.

## Data Availability

The original data are available for reasonable request to Dr Zhao (zhaojingssmu@163.com) and Dr Liu (RenYu.Liu@pennmedicine.upenn.edu).
